# Clinical, Pathologic, and Genetic Spectrum of Collagen VI–Related Disorder in China—A Retrospective Observational Multicenter Study

**DOI:** 10.1155/2024/3503253

**Published:** 2024-10-14

**Authors:** Chaoping Hu, Yiyun Shi, Lei Zhao, Wenhua Zhu, Kexin Jiao, Lifei Yu, Xihua Li, Yi Wang

**Affiliations:** ^1^Department of Neurology, Children's Hospital of Fudan University, Shanghai, China; ^2^National Children's Medical Center, Shanghai, China; ^3^Department of Neurology, Huashan Hospital, Fudan University, Shanghai, China; ^4^Huashan Rare Disease Center, Huashan Hospital, Fudan University, Shanghai, China; ^5^National Center for Neurological Disorders (NCND), Shanghai, China

**Keywords:** Bethlem myopathy, Collagen VI, genotype, phenotype, Ullrich congenital muscular dystrophy

## Abstract

**Background**: Collagen VI-related disorder (COLVI-RD) is one of the most common congenital muscular dystrophies. However, data is limited in China.

**Methods**: We conducted a retrospective study at two tertiary centers. Clinical presentations, lab findings (including serum creatine kinase levels), muscle biopsy, and molecular test results for patients diagnosed with definite COLVI-RD were collected.

**Results**: A total of 82 patients were enrolled in the study, including 4 with early–severe Ullrich congenital muscular dystrophy (E–S UCMD) (4.8%), 45 with moderate–progressive Ullrich congenital muscular dystrophy (M–P UCMD, 54.9%), 19 with mild UCMD (23.2%), and 14 with Bethlem myopathy (BM, 17.1%). Feeding difficulty, DDH, and neurogenic damage were more common in E–S and M–P UCMD, while contracture of distal joints, atrophic scars, and hyperkeratosis was more prominent in mild UCMD and BM. Seventy patients harbored 64 pathogenic mutations in COLVI-related genes: 28 patients in COL6A1 gene, 25 patients in the COL6A2 gene, and 17 patients in the COL6A3 gene, among which 33 mutations were novel. Missense and splicing mutations were predominant for COL6A1 and COL6A3 genes, which were mostly located in N-terminus of THD, in a dominant pattern, while mutations in the COL6A2 gene were much more polymorphic, which spread throughout the whole length of the gene, in a dominant or recessive pattern. Immunofluorescence dual labeling of Collagen VI/IV in 44 patients showed complete deficiency of Collagen VI in 10 patients (22.7%), sarcolemma-specific Collagen VI deficiency in 25 patients (56.8%), and normal Collagen VI staining in 9 patients (20.5%).

**Conclusion**: Our study reported the largest cohort of COLVI-RD in China, which showed M–P UCMD was the most common phenotype, followed by mild UCMD and BM. We identified 30 novel mutations and expanded the genetic spectrum. Missense and splicing mutations were predominant for COL6A1 and COL6A3 genes, while mutations in the COL6A2 gene were much more polymorphic. For severe phenotypes, most mutations are sporadic, while some are AD or recessive inherited. For milder phenotypes, sporadic and AD inherited were both common, while only 1 patient with recessive mutations was observed.

## 1. Introduction

Collagen VI-related disorder (COLVI-RD) is among the most common and accounts for up to 20%–30% of patients with congenital muscular dystrophy worldwide [1–4]. This disorder forms a clinical continuum spectrum characterized by muscle weakness, hypotonia, laxity of distal joints, contractures, and skeletal deformities. The spectrum ranges from the mildest form known as Bethlem myopathy (BM, MIM 158810) to the most severe form known as Ullrich congenital muscular dystrophy (UCMD) (MIM 254090). Intermediate phenotypes (IM) that include limb-girdle muscular dystrophy and myosclerosis myopathy have also been described [5, 6]. The clinical boundaries between these phenotypes have become increasingly blurred as more overlapping cases have been reported. Some studies classified early onset COLVI-RD in the first 2 years of life into early–severe UCMD (E–S UCMD), moderate–progressive UCMD (M–P UCMD), and mild UCMD [1], as important complementation. However, it is still difficult to distinguish with the three or four categories, especially for children in an early stage who were making progress or remained ambulant.

Collagen VI is an extracellular matrix protein that associates closely with the basement membrane of skeletal muscles and links it to the surrounding extracellular matrix. It is made up of three Collagen VI subunits, *α*1, *α*2, and *α*3 chains, which are encoded by the *COL6A1*, *COL6A2*, and *COL6A3* genes, respectively. Each chain contains a central triple helical domain (THD) with a triple Gly-X-Y amino acid sequence, flanked by the N- and C-terminal globular domains [2]. Autosomal dominant BM and de novo dominant UCMD are the most common types of COLVI-RD [1, 3], but rare autosomal recessive BM cases have also been reported in recent years [4, 5]. Among mutations, substitutions of glycins in THD are the most frequent, accounting for 30% of known pathogenic alleles, followed by splice site mutations [6, 7]. While most dominant negative variants have been identified in the N-terminal of the THD, variants of the recessive cases have been found near the C-terminal of THD or in the C-terminal domain. These variants disrupt the initial formation of monomers, thereby preventing their inclusion further in the assembly process [8]. However, exceptions are sometimes described, and the correlation between phenotype and genotypes remains unclear.

In China, only one cohort study of 60 patients with COLVI-RD from North China and one case series of 13 patients from Hong Kong have been reported till now [3, 9, 10]. According to these reports, missense mutations were most common in Chinese COLVI-RD, followed by splicing that cause in-frame exon skipping, small in-frame deletions, and variants leading to premature termination codons (PTCs). No hotspots were identified. The aim of our study is to analyze the genotypes, phenotypes, and pathological features of COLVI-RD and explore their correlations. We aimed to add more data and provide a better understanding of COLVI-RD in the entire Chinese population.

## 2. Patients and Methods

### 2.1. Patients

Patients who exhibited clinical compatibility with COLVI-RD and were confirmed through molecular results and/or muscle biopsy were enrolled, from two neuromuscular centers in China (Children's Hospital of Fudan University and Huashan Hospital of Fudan University) between July 2011 and August 2023. Clinical features included muscle weakness and/or developmental delay, as well as skin changes such as follicular hyperkeratosis and atrophic scar, joint changes including variable proximal contractures and distal hyperlaxity, and congenital hip dislocations. The demographic and clinical data of the patients were reviewed, including information regarding sex, birth, family history, age of onset, best achieved motor function, age of independent walking, motor regression and loss of ambulation, congenital hip dislocation, and torticollis, feeding difficulty, limb–girdle muscle weakness, proximal joint contractures, distal hyperlaxity, scoliosis and other spine problems, related orthopedic surgeries, skin abnormalities, and results of serum creatine kinase and electromyography tests.

The study was approved by the Ethics Committees of Children's Hospital and Huashan Hospital of Fudan University. Informed consent was obtained from all patients.

### 2.2. Categorization of Clinical Phenotype

We divided patients into 4 groups based on their maximal motor capacity, clinical severity and progression, and the situation at the last visit: Patients who never acquired walking ability or who did not acquire motor any milestones at the last visit (> 6 months) were categorized as early–severe type; patients were categorized as moderate–progressive type, encompassing those patients who had delayed motor milestone of unassisted walking and obvious muscle weakness and/or lost ability of independent ambulant before 12 years old; patients who acquired normal motor milestones of ability to walk, developed muscle weakness and lost ability of independent ambulant between 12 and 18 years old were classified into mild UCMD; patients who acquired normal motor milestones of ability to walk, developed mild muscle weakness, and remained independent ambulant after 18 years old were classified to BM. Those patients who cannot be categorized according to the above rules were diagnosed as undefined group (due to loss of follow-up or other special situations).

### 2.3. Muscle Biopsy and Immunofluorescence Analysis of Collagen VI

Muscle biopsy was performed on 9 patients from Huashan Hospital and 38 patients from Children's Hospital according to standard procedures and were then snap frozen by immersion in liquid nitrogen–cooled isopentane. Staining was performed with hematoxylin and eosin, modified Gomori trichrome (MGT), succinate dehydrogenase (SDH), cytochrome c oxidase (COX), and oil red O. Collagen VI labeling was performed using a mouse Anticollagen VI primary antibody (Chemicon, MAB1944) at 1:1000 dilution and Alexa Fluor 555 goat anti-mouse immunoglobulin (Molecular Probes, Eugene, OR) at 1:150 dilution. Basement membrane labeling was performed using a rabbit Anticollagen IV (Abcam, ab6586) at 1:100 dilution and Alexa Fluor 488 goat anti-rabbit IgG (Molecular Probes, Eugene, OR) at 1:500 dilution. Images were obtained using a NikonECLIPSE-80I microscope. Muscle biopsies were also analyzed by immunofluorescence for dystrophin expression with each of the three antibodies targeting NCL-DYS1, NCL-DYS2, and NCL-DYS3 (Leica Biosystems Newcastle), as well as the detection of dysferlin (NCL-Hamlet, Leica), *α*-sarcoglycan (05–593, Millipore), *β*-sarcoglycan (NCL-b-SARC, Leica), *γ*-sarcoglycan (NCL-g-SARC, Leica), *δ*-sarcoglycan (NCL-d-SARC, Leica), and Collagen VI (MAB1944, Millipore).

### 2.4. Mutation Analysis

Molecular tests were conducted on 14 patients from Huashan Hospital and 58 patients from the Children's Hospital of Fudan University. Genomic DNA was extracted from blood using standard procedures. The DNA samples were screened for targeted next-generation sequencing (NGS) of genes related to neuromuscular diseases. All the mutations identified by NGS were subsequently confirmed by Sanger sequencing. Sequences were analyzed according to the reference sequence of Collagen VI genes in the GenBank database (gene identification: *COL6A1*, NM_001848.2; *COL6A2*, NM_001849.3; *COL6A3*, NM_004369.3). Homologene was used to compare nucleotide sequences between multiple organisms to assess the amino acid conservation.

RNA was extracted from the muscle tissue of 4 patients from Huashan Hospital and retrotranscribed to cDNA using standard procedures. cDNA was sequenced to assess the pathogenicity of the potential splicing mutations.

## 3. Results

### 3.1. Patients and Demographics

Our cohort enrolled 82 patients with a confirmed diagnosis of COLVI-RD. All of them were of Chinese Han origin. Seventy-two patients performed molecular tests, and 70 patients were confirmed with mutations in Collagen VI-related genes, including 35 patients who also conducted muscle biopsy and were diagnosed with both molecular and pathological results. Two patients revealed no pathogenic mutations and were diagnosed by muscle biopsy and immunofluorescence staining. The rest 10 patients did not receive molecular tests (their parents denied or molecular tests were unavailable at the time) and were diagnosed merely by muscle biopsy and immunofluorescence staining from muscle tissues. Of 82 patients, 49(59.8%) were male, and 33 (40.2%) were female. The age of the first evaluation ranged from 0.1 years to 17 years (median: 5.0 years), while the onset of age ranged from birth to 11.6 years old (median: 1.5 years). The age of the last visit ranged from 0.3 to 44 years old (median: 8 years old). Twenty-five patients were evaluated once only, and the median follow-up duration of the rest patients was 4 years (ranging from 0.1 to 10.8 years). Of them, 14 patients had a family history (Pt40 and Pt41 were siblings).

### 3.2. Phenotype and Clinical Classification

**Figure 1:**
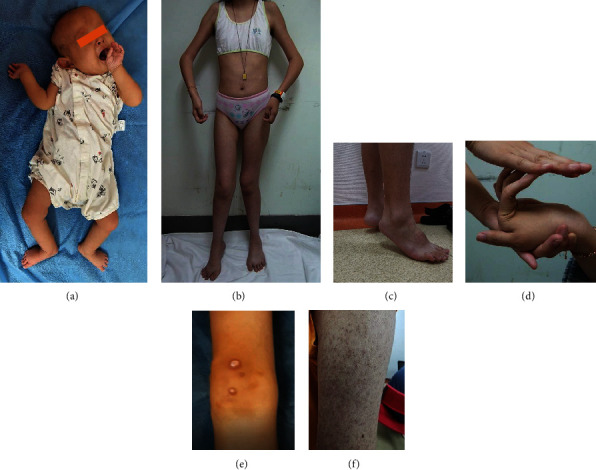
Clinical pictures of different phenotypes of COLVI-RD. (a) Severe proximal contractures and distal hyperlaxity since birth and torticollis (E-S UCMD). (b–d) Proximal contractures and distal hyperlaxity and Achilles tendon contracture in M–P UCMD. (e, f) Atrophic scars and hyperkeratosis of the skin mainly in mild UCMD and BM.

Patients were divided into 4 groups: 4 with early sever Ullrich congenital muscular dystrophy (E–S UCMD) (4.8%), 45 with moderate–progressive Ullrich congenital muscular dystrophy (M–P UCMD, 54.9%), 19 with mild UCMD (23.2%), and 14 with Bethlem myopathy (BM, 17.1%) (Table 1). Distal hyperlaxity (82.6%), distal contracture (59.6%), and proximal contracture (46.2%) were most common in COLVI-RD, followed by atrophic scars (33.3%) and hyperkeratosis (25.8%) (Figure 1). Twelve patients had a family history. The details of clinical presentations and lab findings of 82 patients are shown in Table S1 (see Supporting Information).

### 3.3. Collagen VI Expression in Muscle Biopsies

**Figure 2:**
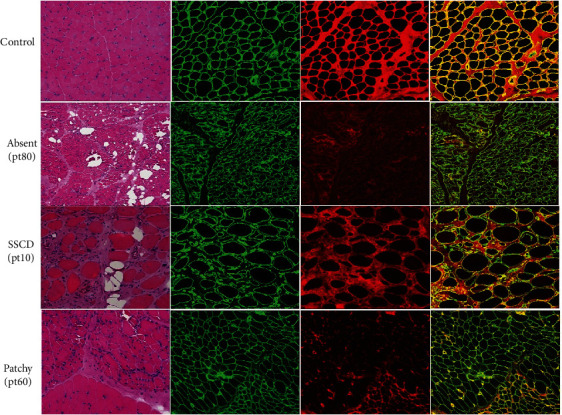
Pathological findings in COLVI-RD. Dual immunofluorescence of Collagen VI (red) and Collagen IV (green) showed colocalization on the basement membrane (yellow) mimicked in the control case or complete deficiency (absent, pt80) or poor colocalization on the basement membrane (SSCD, pt10) or patchy deficiency (pt60). Scale bar is 100 *μ*m.

In total, 47 patients received muscle biopsy. From HE staining, 39 patients revealed muscular dystrophic change, 4 patients showed chronic myopathy change, 3 patients showed normal, and 1 patient showed neurogenic damage change. Immunofluorescence of dual labeling of Collagen VI/IV in 44 patients indicated complete deficiency of Collagen VI in 10 patients, sarcolemma-specific Collagen VI deficiency in 25 patients, and normal Collagen VI staining in 9 patients (Figure 2).

### 3.4. Molecular Tests

**Figure 3:**
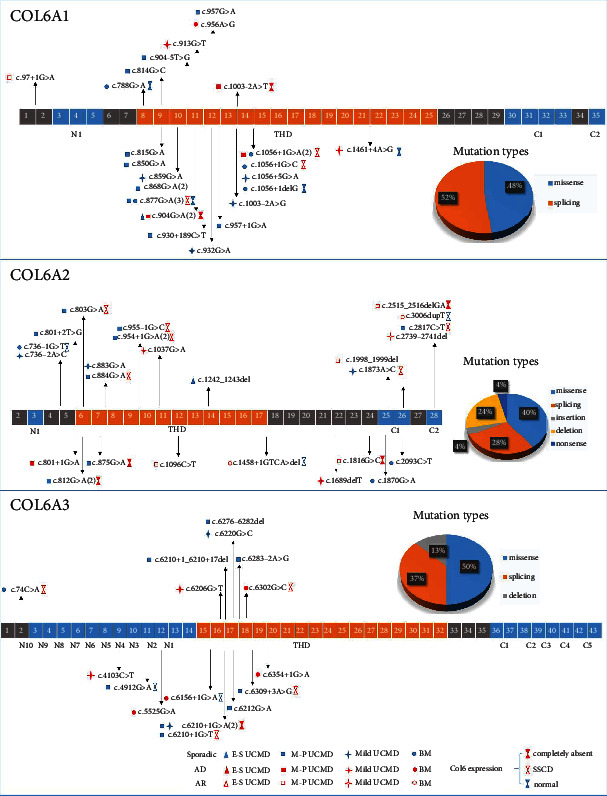
Schematic localization of the mutations found in the *α*1, *α*2, and *α*3 chains of Collagen VI. Exons are shown as boxes. The domains are indicated below the diagram (THD: triple helical domain; N10-N15: N-terminal domains; C1-C5: C-terminal domains). Novel and reported mutations are indicated above or below the genes, respectively. Symbols in front of the mutations refer to the clinical phenotype of the patients: Triangles were early–severe UCMD patients, squares were moderate–progressive UCMD patients, stars were mild UCMD, and circles were BM patients. Dominant mutations were marked in red and sporadic in blue, while recessive mutations were hollow red. Hourglass symbols refer to the expression of Collagen VI in pathogenic features of the patients: Solid red indicated completely absent, hollow red was SSCD, and hollow blue was normal.

In our study, 70 patients (70/72, 93.2%) harbored 64 pathogenic mutations in Collagen VI-related genes: 28 patients in the *COL6A1* gene, 25 patients in the *COL6A2* gene, and 17 patients in the *COL6A3* gene, among which 33 mutations were novel (Figure 3, Table 2). Of them, missense mutations were most common (29, 45.3%), followed by splicing mutations (25, 39.1%), deletion mutations (8, 12.5%), and nonsense mutations (2, 3.1%). Forty-nine mutations were located in the THD domain (69.0%), 15 mutations were located in the C-terminal region (21.1%), and 7 mutations were in the N-terminal region (10.9%). For missense mutations, 20 were glycine substitutions in the N-terminal region of the TH domain that interrupt the Gly-X-Y amino acid motif (20/29, 67.0%). The following mutations were recurrent: c.877G>A(p.G293R), c.868G>A(p.G290R), c.1056+1G>A and c.904G>A(p.G302R) in *COL6A1* gene, c.812G>A(p.G271D) (in 2 compound heterozygous patients) and c.954+1G>A in *COL6A2* gene, and c.6210+1G>A in *COL6A3* gene. The mutations identified were summarized in Table S2 (see Supporting Information).

#### 3.4.1. Mutation Identification in the *COL6A1* Gene

A total of 23 mutations in 28 patients were identified in the *COL6A1* gene, among which 7 mutations were novel and 5 patients had a family history. Of the 23 mutations found, 12 were splicing mutations, and 11 were missense mutations.

#### 3.4.2. Mutation Identification in the *COL6A2* Gene

A total of 25 mutations in 25 patients were identified in the *COL6A2* gene, including 10 missense mutations, 7 splicing mutations, 6 deletions, 1 insert mutation, and 1 nonsense mutation. Among them, 17 mutations were novel. Five patients had a family history (pt42 and pt43 were twin sisters).

#### 3.4.3. Mutation Identification in the *COL6A3* Gene

A total of 16 mutations in 17 patients were identified in the *COL6A3* gene, among which 9 mutations were novel. Of the 16 mutations found, 8 were missense mutations, 6 were splicing mutations, and 2 were deletion mutations. Five patients had a family history.

#### 3.4.4. RT-PCR Analysis of Four Patients With COLVI-RD

**Figure 4:**
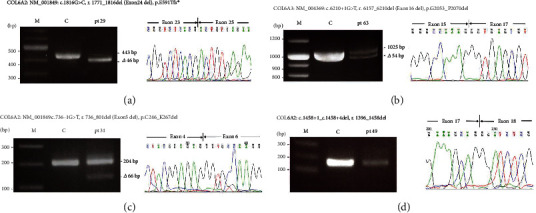
RT–PCR in pt29, pt31, pt63, and pt49. (a) RT–PCR of a2(VI) bases showing the normal 443 bp product and the 397 bp product amplified from pt29 mRNA, while direct sequencing demonstrated that the smaller product lacks the bases encoded by *COL6A2* exon 24. (b) RT-PCR of a3(VI) bases showing the normal 1025 bp product and the 971 bp product amplified from pt63 mRNA, while direct sequencing demonstrated that the smaller product lacks the bases encoded by *COL6A3* exon 16. (c) RT-PCR of a2(VI) bases showing the normal 204 bp product and the 138 bp product amplified from pt31 mRNA, while direct sequencing demonstrated that the smaller product lacks the bases encoded by *COL6A3* Exon 5. (d) RT-PCR of a2(VI) did not show the difference in molecular weight of the product amplified from pt49 mRNA but showed a markedly reduced amount of mRNA RT-PCR product. Direct sequencing revealed no exon skipping caused by mutation c.1458+1_c.148+4del, and another mutation c.3006dupT in pt49 created a premature stop codon (as shown in Table S2).

RT-PCR analysis revealed Exon 24 skipping in mRNA of the *COL6A2* gene in pt29, Exon 16 skipping in pt63, and Exon 5 skipping mRNA of the *COL6A3* gene in pt31, respectively. RT-PCR of a2(VI) did not show the difference in molecular weight of the product amplified from pt49 mRNA but showed a markedly reduced amount of mRNA RT-PCR product (Figure 4).

#### 3.4.5. SV/CNV/UPD Test

Trio WES in patients 41 and 42 revealed homozygous c.1037G>A variant in *COL6A2* with maternal origin. Thus UPD test was performed, indicating a 21.16 Mb uniparental disomy in q21.3q22.3, which originated from her mother and included the *COL6A2* gene.

## 4. Discussion

### 4.1. Flag Symptoms of Severe Phenotype

Clinical heterogeneity and overlapping between different subphenotypes of COLVI-RD is remarkable in our cohort, similar to previous reports [11]. Sometimes, it is still difficult to differentiate in early age. From our cohort, feeding difficulty and DDH were more common in severe forms, which was the same situation for neurogenic damage in the EMG test and complete deficiency of collagen VI in IHF. In contrast, contracture of distal joints, atrophic scars, and hyperkeratosis were more prominent in milder patients. Larger cohort studies of multicenter studies are needed for further investigation.

### 4.2. Genotype Spectrum of COLVI-RD in China

In our cohort, patients with mutations in the *COL6A1* gene and *COL6A2* gene were more common than the *COL6A3* gene. And missense and splicing mutations were most common in *COL6A1* and *COL6A3* mutated patients, consistent with previous studies [12, 13]. In the *COL6A1* gene, the majority of mutations were located between Exons 8 and 14 of the THD domain, among which missense mutations in Exon 10 and splicing mutations in Intron 14, which caused in-frame Exon 14 skipping, were most common. Sporadic (de novo dominant) mutations were most common (75.0%), followed by mutations inherited in AD pattern (21.4%). In the *COL6A3* gene, most mutations were missense and splicing mutations located between Exon 15 and Exon 19 of the THD domain, followed by missense mutations located in the N-terminus of THD. Among them, splicing mutations leading to Exon 16 skipping and missense mutations in Exon 17 were most seen, indicating the important roles of Exons 16 and 17 in the *α*3 chain. In *COL6A3*, de novo dominant mutations were most common (64.7%), followed by mutations inherited in AD pattern (35.3%). Only 1 patient with homozygous mutations in the signal peptide of the *COL6A1* gene was observed in our cohort, who presented with M–P UCMD.

Different from *COL6A1* and *COL6A3*, mutations in the *COL6A2* gene were much more polymorphic; although missense and splicing mutations were still most common (40% and 28%), deletion was not insignificant (24%). Additionally, mutations were observed throughout the whole length of the *COL6A2* gene, from the N-terminus (16.0%) to THD (44.0%) and C-terminus (40.0%). One study reported that most dominant negative variants in the *COL6A2* gene were identified in the N-terminal of THD, while variants of the recessive cases were found near the C-terminal of THD or in the C-terminal domain [10]. In our cohort, mutations in the N-terminal were dominant negative, but both dominant and recessive mutations were observed in THD and C-terminal. It is special in 3 Collagen VI encoding genes, and we suppose that it may be related to the unique role of the *COL6A2* gene in the assembly and function of Collagen VI; the underlying mechanism of each domain is still to be studied.

### 4.3. Association Between Phenotype and Genotype

Homozygous PTC–causing mutations in the triple helix domains were reported to usually cause E-S UCMD [1]. Differently, in our cohort, two patients with E-S UCMD revealed a missense p.G302R mutations in *COL6A1* (pt 11) and a frame-shift p.G415Afs^∗^15 mutation in *COL6A2* gene (pt 53). Both of them were dominant negative, one sporadic and one AD inherited. However, the father of patient 53, who carried the same mutation, only presented with slight hyperkeratosis. Somatic mosaicism might be the explanation.

M–P UCMD, which is the classical UCMD form, was the most common phenotype in our cohort. Most patients were dominant sporadic (78.4%), followed by AD inherited (10.8%) and recessive inherited (10.8%). In *COL6A1* and *COL6A3* gene mutations, glycine substitutions toward the N terminus of the triple helix were reported to be more frequently seen in severe phenotypes including E–S UCMD and M–P UCMD [14]. In our study, for the *COL6A1* gene, glycine substitutions toward the N terminus (Exons 9–12) mainly caused M–P UCMD while splicing mutations in the C-terminal of THD (Exons 13–15) mostly led to milder forms. In contrast, for the *COL6A2* gene, both dominant and recessive mutations were observed. Most recessive mutations were deletion or missense mutations and were located in C-terminal THD or C-terminus, while almost dominant negative mutations were splicing or glycine substitutions in THD and N-terminals.

In the mild UCMD group, the percentage of sporadic and AD-inherited patients was close (47.1% vs. 41.7%). Only two twin-sister patients carried homozygous missense p.G346E mutations in the *COL6A2* gene. Further molecular test revealed maternal UPD, which was an uncommon phenomenon in COLVI-RD and has not been reported before.

In BM groups, most mutations were sporadic (8/13, 61.5%), while some were AD inherited (4/13, 30.8%). The only BM patient with recessive mutations harbored a reported c.1458+1_c.1458+4del mutation in the THD domain and a novel c.3006dupT(p.D1003^∗^) mutation in the C2 domain in *COL6A2*. The mutation c.1458+1_c.1458+4del was reported in a dominant BM patient, causing disorganized and rarefied collagen VI IF on fibroblasts but immunohistochemistry finding was normal in muscle biopsy [14]. While the cDNA sequencing did not reveal a splicing effect, the amount of cDNA PCR product was markedly reduced in our patient (Figure 4(d)). He had normal collagen VI expression on muscle biopsy and presented with BM, a relatively milder phenotype. It seemed that the dominant negative effect may not be the only pathophysiological mechanism, and decreased production of*α*2 chain may impose additive effect for the clinical presentations of these two patients.

Intra and interfamiliar phenotype variation was observed in some pedigrees, like the previous report [6]. As one of the hot spot mutations in the *COL6A1* gene, c.868G>A(p.G290R) was reported to cause varied phenotypes ranging from UCMD to BM [8, 15–17]. In our study, 3 patients who carried the same heterozygous c.877G>A(p.G293R) mutation in the *COL6A1* gene presented from severe UCMD to mild BM phenotype. What is more, intrafamiliar phenotype variability was common. Six parents who carried the same mutation with the index patients, presented with very mild or even no symptoms. Some experts proposed somatic mosaicism as one potential hypothesis [18], but the underlying mechanism still remains to be elucidated. Clinical severity and mode of inheritance of COLVI-RD are believed to be linked to functional consequences of mutations affecting Collagen VI genes as well as an additional genetic burden, which forms the context of mutation expressivity [7].

### 4.4. Association Between Pathology and Genotypes

The pathology in our patients of COLVI-RD revealed a broad spectrum of change, varying from severe dystrophic change to even normal results in HE staining, consistent with previous reports [19]. Meanwhile, dual immunofluorescence labeling of collagen VI with collagen IV showed a broad spectrum of expression of collagen VI, ranging from normal to completely absent. Consistent with previous reports [1], in our cohort, complete deficiency of Collagen VI was mostly observed in severe UCMD patients, as well as in some milder phenotypes. Like previous report [20], Collagen VI showed normal in most AD-inherited BM patients, but one patient with compound heterozygous mutations was also noted, which indicated that besides the dominant inheritance pattern, there may be other factors playing certain roles for this phenomenon. Interestingly, we observed patchy deficiency of collagen VI in a BM patient with heterozygous c.74C>A(p.A25D) mutation in the signal peptide of *α*3 chain, indicating that normal and mutant transcript coexisted in the patient with some unknown mechanism, which needed further investigation. However, there seemed no clear correlation between the degree of collagen 6 expression in immunofluorescent labeling with either the mutation type or domain. Complete deficiency of Collagen VI was observed in splicing, transition, or frameshift mutations in all three Collagen VI genes. Interestingly, some patients with the same mutations presented with different levels of expression of Collagen VI, and one patient even showed a patchy deficiency of Collagen VI in one muscle bundle, which indicated other factors interfering with the expression of Collagen VI in COLVI-RD.

The limitations of our study were that motor function scales such as MFM, degrees of contractures, spinal deformity, and the effect of surgical intervention were not included. And further investigation is needed in prospective studies [21]. Another limitation of our study was that immunofluorescent labeling of Collagen VI in fibroblast cultures was not performed, which was proposed as a useful tool for basal diagnostic services for BM [14].

## 5. Conclusion

This was the largest cohort study of COLVI-RD in China. M–P UCMD was the most common phenotype, followed by mild UCMD and BM. Mutations in the *COL6A1* gene were most common, followed by *COL6A2* and *COL6A3*. Dominant missense and splicing mutations were predominant for *COL6A1* and *COL6A3* genes, while mutations in the *COL6A2* gene were much more polymorphic. For severe phenotypes, most mutations were dominant sporadic, while some were AD or recessive inherited. For milder phenotypes, sporadic and AD inherited were both common, while only 1 patient with recessive mutations was observed.

## Figures and Tables

**Table 1:** The clinical and genetic characteristics of 82 COLVI-RD patients.

/	/	**Total (** **n** ** = 82)**	**E**–**S UCMD (n = 4)**	**M**–**P UCMD (n = 45)**	**Mild UCMD (** **n** ** = 19)**	**BM (** **n** ** = 14)**
Sex	Male:female	49:33	2:2	27:18	13:6	7:7

Clinical symptoms	Mean onset of age (median) (ys)	1.7 (range: 0.1–11.6)	0.3 (range: 0.1–1.5)	1 (range: 0.1–5)	3 (range: 0.1–7.5)	2 (range: 0.1–11.6)
	Median age of walking (ys)	1.5 (range: 1 ~ 5)	NA	1.5 (range: 1~5)	1.0 (range: 1~1.3)	1.0 (range: 1~5)
	Median age of lost ambulance (ys)	8 (range:3~44)	NA	7 (range: 3~11)	16 (range: 13~16)	26 (range: 18–44)

Clinical symptoms	Distal hyperlaxity (%)	57/69 (82.6)	3/4(75.0)	36/39 (92.3)	13/14 (92.9)	5/12 (41.7)
	Proximal contracture (%)	31/67 (46.2)	1/4 (25.0)	19/38 (50.0)	3/13 (23.1)	8/12 (66.7)
	DDH (%)	19/69 (27.5)	2/4 (50.0)	15/39 (38.5)	2/14 (14.3)	0/12 (0)
	Distal contracture (%)	34/57 (59.6)	0/4 (0)	15/31 (48.4)	9/12 (75.0)	10/10 (100.0)
	Torticollis (%)	13/70 (18.6)	2/4 (50.0)	8/40 (20.0)	0/14 (0)	3/12 (25.0)
	Feeding difficulty (%)	14/75 (18.7)	3/4(75.0)	6/40 (15.0)	3/19 (15.8)	2/12 (16.7)
	Hyperkeratosis (%)	17/66 (25.8)	0/4 (0)	6/37 (16.2)	3/12 (25.0)	8/13 (61.5)
	Atrophic scars (%)	22/66 (33.3)	1/4 (25.0)	9/37 (24.3)	3/12 (25.0)	9/13 (69.2)

Creatine kinase (IU/L)	Mean(SD)	366.7 (216.0)	124.7 (69)	340.3 (187.6)	431.1 (236.1)	392.2 (236.1)

EMG	Neurogenic damage	5	0	5	0	0
	Myogenic damage	31	2	17	12	9
	Mixed damage	14	1	8	4	1
	Normal	4	1	2	1	0

IHF of Collagen VI in muscle	Complete deficiency	10	1	1	8	0
	Sarcolemma specific	25	1	3	16	5
	Normal expression	8	0	1	2	5

**Table 2:** Distribution and pathogenic criteria (ACMG) of 32 novel mutations in our cohort.

**No.**	**Gene**	**Exon**	**Domain**	**Nucleotide change**	**Hom/het**	**Amino acid change**	**Mutation type**	**ACMG**	**Criteria**	**Origin**	**Mechanism of the allele**
P4	COL6A1	In1	N	c.97+1G>A	Hom	/	Splicing	LP	PVS1 + PM2 + PP4	NA	AR
P10	COL6A1	In13	THD	c.1003-2A>T	Het	/	Splicing	LP	PVS1 + PM2 + PP4	Maternal	AD
P15	COL6A1	Ex11	THD	c.913G>T	Het	p.G305W	Missense	LP	PM1 + PM2 + PP1 + PP2 + PP3 + PP4	Maternal	AD
P18	COL6A1	In10	THD	c.904-5T>G	Het	/	Splicing	LP	PS2 + PM2 + PP4	De novo	AD
P19	COL6A1	E12	THD	c.957G>A	Het	/	Splicing	LP	PS2 + PM2 + PP4	De novo	AD
P20	COL6A1	Ex9	THD	c.814G>C	Het	p.G272R	Missense	P	PS2 + PM1 + PM2 + PM5 + PP2 + PP3 + PP4	De novo	AD
P26	COL6A1	Ex8	THD	c.788G>A	Het	p.G263D	Missense	P	PS2 + PM1 + PM2 + PM5 + PP2 + PP3 + PP4	De novo	AD
P31	COL6A2	In4	THD	c.736-1G>T	Het	p.C246_K267del	Splicing	P	PVS1 + PM2 + PM6 + PP4	NA	AD
P32	COL6A2	Ex7	THD	c.883G>A	Het	p.G295R	Missense	LP	PM1 + PM2 + PM6 + PP2 + PP3	NA	AD
P35	COL6A2	In9	THD	c.954+1G>A	Het	/	Splicing	P	PVS1 + PS2 + PS4 + PM2 + PP4	De novo	AD
P36	COL6A2	In9	THD	c.954+1G>A	Het	/	Splicing	P	PVS1 + PS2 + PS4 + PM2 + PP4	De novo	AD
P37	COL6A2	In9	THD	c.955-1G>C	Het	/	Splicing	P	PVS1 + PS2 + PM2 + PP4	De novo	AD
P38	COL6A2	In4	N1	c.736-2A>C	Het	/	Splicing	P	PVS1 + PS2 + PM2 + PP4	De novo	AD
P39	COL6A2	In5	THD	c.801+2T>G	Het	/	Splicing	P	PVS1 + PS2 + PM2	De novo	AD
P40	COL6A2	Ex28	C2	c.2817C>T	Het	p.H939H	Missense	VUS	PM6 + PP4	NA	AD
P41	COL6A2	E11	THD	c.1037G>A	Hom	p.G346E	Missense	LP	PM2 + PM3 + PP1 + PP3 + PP4	Maternal	AR
P42	COL6A2	E11	THD	c.1037G>A	Hom	p.G346E	Missense	LP	PM2 + PM3 + PP1 + PP3 + PP4	Maternal	AR
P44	COL6A2	Ex7	THD	c.884G>A	Het	p.G295E	Missense	LP	PM1 + PM2 + PM5 + PP3 + PP4	NA	AD
P46	COL6A2	Ex25	C1	c.1873A>C	Het	P.S625R	Missense	P	PS4_moderate+PM2 + PP1 + PP3 + PP4	Paternal	AD
P47	COL6A2	Ex28	C2	c.2739-2741del	Het	p.914Fdel	Deletion	LP	PM2 + PM3 + PM4 + PP4	Maternal	AR
		Ex22	C1	c.1689delT	Het	p.G564Vfs^∗^32	Deletion/frame shift	LP	PVS1 + PM2 + PP4	Paternal	AR(reported)
P48	COL6A2	Ex6	THD	c.803G>A	Het	p.G268D	Missense	LP	PM1 + PM2 + PM5 + PP3 + PP4	De novo	AD
P49	COL6A2	Ex28	C2	c.3006dupT	Het	p.D1003^∗^	Duplication	P	PVS1 + PM2 + PM4 + PP4	NA	AR
		In17	THD	c.1458+1_c.1458+4del	Het	p.G466_ E486del	Deletion	P	PVS1 + PM2 + PM4 + PP4	NA	AR(reported)
P51	COL6A2	Ex25	C1	c.1998_1999del	Het	p.S66Rfs^∗^5	Deletion/frame shift	P	PVS1 + PM2 + PM3 + PP4	Maternal	AR
		E12	THD	c.1096C>T	Het	p.R366X	Nonsense	P	PVS1 + PS4 + PM2 + PP4	Paternal	AR(reported)
P52	COL6A2	Ex28	C2	c.2515_2516delGA	Hom	p.D839Rfs^∗^7	Deletion/frame shift	P	PVS1 + PM2 + PM3 + PP4	Parental	AR
p53	COL6A2	Ex14	THD	c.1242_1243del	Het	p.G415Afs^∗^15	Deletion/frame shift	P	PVS1 + PM2 + PM3 + PP4	Paternal	AD^a^
P56	COL6A3	In17	THD	c.6283-2A>G	Het	/	Splicing	P	PVS1 + PM2 + PM3 + PP4	De novo	AD
P57	COL6A3	Ex17	THD	c.6276-6282del	Het	p.V2093A fs^∗^11	Deletion/frame shift	P	PVS1 + PM2 + PM3 + PP4	De novo	AD
P60	COL6A3	Ex2	SP	c.74C>A	Het	p.A25D	Missense	LP	PS2 + PM2 + PP3 + PP4	De novo	AD
P61	COL6A3	Ex16	THD	c.6206G>T	Het	p.G2068C	Missense	LP	PM1 + PM2 + PP1 + PP2 + PP3 + PP4	Maternal	AD
P64	COL6A3	Ex18	THD	c.6302G>C	Het	p.G2101A	Missense	LP	PM1 + PM2 + PP1 + PP2 + PP3 + PP4	Maternal	AD
P66	COL6A3	Ex17	THD	c.6220G>C	Het	p.G2074R	Missense	P	PS2 + PM1 + PM2 + PM5 + PP2 + PP3 + PP4	De novo	AD
P67	COL6A3	In16	THD	c.6210+1_6210+17del	Het	/	Deletion	LP	PS2 + PM1 + PM2 + PP4	De novo	AD

Abbreviation: SP: signal peptide.

^a^AD: This patient's father/mother was an asymptomatic carrier.

## Data Availability

The data used to support the findings of this study are available from the corresponding author upon request.
