# Supplementary material for: Clinical, Pathologic, and Genetic Spectrum of Collagen VI–Related Disorder in China—A Retrospective Observational Multicenter Study
Source: Hum Mutat. 2024 Oct 14;2024:3503253. doi: 10.1155/2024/3503253 (PMC11918884; doi:10.1155/2024/3503253)
Supplement: Supporting Information — Additional supporting information can be found online in the Supporting Information section. See Table S1 and Table S2 for comprehensive clinical and genetic data analysis. [file 3503253.f1.docx]

S-Table 1. Clinical features of 82 collagen VI-related myopathy patients

| No. | phenotype | sex | age of first visit | age at last visit(ys) | age of walking(ys) | feeding difficulty | age of onset | Max motor ability | age of loss of ambulation(ys） | Musculoskeletal | | | | | | skin | | CK (IU/L) | EMG | surgery |
| --- | --- | --- | --- | --- | --- | --- | --- | --- | --- | --- | --- | --- | --- | --- | --- | --- | --- | --- | --- | --- |
|  |  |  |  |  |  |  |  |  |  |  |  |  |  |  |  |  |  |  |  |  |
|  |  |  |  |  |  |  |  |  |  | proximal contracture | distal hyperlaxity | scoliosis | hip dislocation | torticollis | distal contracture | Hyperkeratosis | atrophic scars |  |  |  |
| P1 | Mild | M | 3.3 | 3.7 | 1.3 | + | 1.5 | walk | walking | - | + | - | - | - | NA | NA | NA | 328 | myo | - |
| P2 | M-P | F | 0.8 | 6 | 3 | - | 1 | walk | walking | - | + | - | + | - | NA | - | - | NA | NA | - |
| P3 | Mild | M | 3 | 8.3 | 1.3 | - | 3.5 | walk | walking | - | + | - | - | - | NA | - | - | 364 | myo | - |
| P4 | M-P | m | 1.8 | 5 | 1 | + | 0.5 | walk | 3 | - | + | - | - | - | NA | - | - | 375 | myo | - |
| P5 | Mild | m | 5.1 | 5.4 | 1.3 | + | 1 | walk | walking | - | + | - | - | - | NA | - | - | 616 | myo | - |
| P6 | M-P | f | 0.1 | 9.3 | 1.5 | - | 0.1 | walk | 8 | - | + | - | - | - | NA | - | - | 309 | myo+neu | Tor |
| P7 | M-P | m | 2.8 | 3.1 | 1.5 | - | 0.5 | walk | walking | NA | NA | NA | NA | NA | NA | NA | NA | NA | NA | - |
| P8 | BM | f | 10.1 | 19.3 | 1.3 | - | 2 | walk | walking | + | - | - | - | - | NA | + | + | 484 | NA | AT*2 |
| P9 | BM | f | 8.3 | 17 | 1 | + | 0.1 | walk | walking | - | - | - | - | + | NA | - | - | 832 | myo | AT+Tor |
| P10 | M-P | f | 7.6 | 7.6 | NA | NA | NA | walk | walking | + | - | - | - | - | NA | - | + | 439 | myo | - |
| P11 | E-S | m | 13.1 | 13.5 | NP | - | 1.5 | assisted walk | 7 | - | - | - | - | - | - | - | - | 204 | myo+neu | Tor |
| P12 | M-P | f | 5.3 | 7.9 | NA | - | 3 | walk | walking | - | - | - | + | - | - | - | - | 779 | neu | - |
| P13 | M-P | m | 4 | 14.8 | 1 | - | 3 | walk | walking | - | + | - | - | - | + | + | + | 693 |  | AT |
| P14 | M-P | f | 1.7 | 12.5 | 2 | - | 2 | walk | walking | + | + | + | + | - | + | - | + | 217 | myo | DDH |
| P15 | Mild | m | 5.9 | 5.9 | 1.3 | - | 1.5 | walk | walking | NA | NA | NA | NA | NA | NA | NA | NA | NA |  | - |
| P16 | Mild | m | 2.8 | 8.5 | NA | - | 3 | walk | walking | NA | NA | NA | NA | NA | NA | NA | NA | 287 | neu+myo | - |
| P17 | BM | f | 6.7 | 17 | 1.2 | + | 7 | walk | walking | - | + | + | - | + | + | + | - | 106 | myo | Tor |
| P18 | M-P | m | 13 | 14.9 | 5 | - | 0.4 | walk | walking | + | + | - | + | + | + | NA | + | NA | myo | Mo+Tor |
| P19 | M-P | f | 4 | 4 | 3 | + | 0.1 | walk | walking | + | + | - | + | + | + | - | - | 288 | NA | DDH |
| P20 | M-P | m | 0.9 | 6 | 1.5 | + | 0.5 | walk | walking | - | + | - | - | - | - | + | - | 319 | NA | - |
| P21 | Mild | m | 4.1 | 11 | 1 | - | 4 | walk | walking | - | + | - | + | - | - | - | - | 225 | myo+neu | - |
| P22 | M-P | m | 9.3 | 13 | NA | NA | NA | walk | 8 | + | - | + | - | - | + | - | - | NA | NA | - |
| P23 | BM | f | 10.5 | 17.5 | NA | - | NA | walk | walking | + | - | - | - | - | + | - | - | 401 | myo | - |
| P24 | M-P | f | 4 | 4 | 1.5 | + | 0.1 | walk | walking | + | + | - | + | + | + | + | - | nor | nor | - |
| P25 | M-P? | M | 3 | 3 | 1.5 | - | 0.1 | walk | walking | NA | NA | NA | NA | NA | NA | NA | NA | NA | NA | - |
| P26 | BM | M | 14 | 29 | 1 | - | 2 | walk | 31 | + | + | + | - | - | + | + | + | 184 | myo | AT |
| P27 | M-P | m | 13 | 15 | 3 | - | 1.5 | walk | walking | + | + | - | - | - | - | NA | NA | nor | NA | - |
| P28 | BM | F | 10 | 18 | NA | - | 7 | walk | 18 | + | + | - | - | - | + | - | + | 195 | myo | - |
| P29 | M-P | F | 9 | 11 | 2 | - | 0.1 | walk | 7 | + | + | + | - | + | NA | - | - | 163 | myo | Tor |
| P30 | BM | M | NA | 44 | 1 | NA | NA | walk | 44 | + | - | - | - | - | + | + | + | 452 | NA | - |
| P31 | BM | M | NA | 26 | 1 | NA | NA | walk | 26 | + | + | + | - | - | + | + | + | 152 | myo | - |
| P32 | Mild？ | M | 8 | 8 | NA | - | 7.5 | walk | walking | NA | NA | NA | NA | NA | + | NA | NA | 623 | myo | - |
| P33 | M-P | m | 4.3 | 6.5 | 1.5 | - | 1.5 | walk | walking | - | + | - | - | - | - | - | - | nor | myo | - |
| P34 | BM | m | NA | 16.7 | NA | - |  | walk | walking | NA | NA | NA | NA | NA | + | + | + | NA | NA | - |
| P35 | M-P | f | 2.5 | 6.9 | 1.2 | - | 2.5 | walk | 5 | + | + | - | - | - | - | - | - | 526 | myo | - |
| P36 | M-P | m | 6.1 | 8.1 | 1.5 | - | 3.4 | walk | walking | NA | + | - | - | - | + | + | - | nor | neu+myo | - |
| P37 | M-P | m | 2.3 | 5 | 1.8 | - | 1.5 | walk | walking | - | + | - | - | - | + | - | + | 586 | neu+myo | - |
| P38 | Mild | f | 6 | 14 | 1.3 | + | 2 | walk | walking | + | + | - | - | - | + | + | + | 292 | NA | AT |
| P39 | M-P | m | 2 | 2 | NA | NA | 1.5 | walk | walking | NA | NA | NA | NA | NA | NA | NA | NA | NA | myo | - |
| P40 | M-P | m | 2.1 | 3 | 1.5 | + | 2 | walk | walking | - | + | - | - | - | - | - | - | 293 | neu+myo | - |
| P41 | Mild | f | 5.8 | 5.8 | 1.2 | - | 1.2 | walk | walking | - | + | - | - | - | - | - | - | 767 | myo | - |
| P42 | Mild | f | 5.8 | 5.8 | 1.2 | - | 1.2 | walk | walking | - | + | - | - | - | + | - | - | 767 | myo | - |
| P43 | M-P | f | 3.9 | 8.5 | 2 | - | 0.1 | walk | NA | - | + | - | + | - | + | - | + | NA | myo | DDH |
| P44 | M-P | m | 3.3 | 9 | 1.5 | - | 3.3 | walk | walking | - | + | - | - | - | - | - | - | 376 | myo | - |
| P45 | M-P | f | 5.6 | 5.7 | 1.5 | - | 0.1 | walk | walking | - | + | + | + | + | NA | - | + | 102 | neu+myo | - |
| P46 | Mild？ | m | 3.3 | 6.1 | 1.3 | - | 0.1 | walk | walking | - | + | - | - | - | + | - | - | 699 | myo | - |
| P47 | Mild？ | f | 6.9 | 6.9 | 1.2 | - | 1.5 | walk | walking | NA | NA | NA | NA | NA | NA | NA | NA | 325 | myo | - |
| P48 | M-P | f | 6.5 | 9 | 1.5 | - | 2 | walk | walking | - | + | - | - | - | - | - | - | 332 | NA | - |
| P49 | BM | M | 15 | 19 | 1 | - | NA | walk | 19 | + | + | - | - | - | NA | + | + | 836 | myo | - |
| P50 | M-P | f | 12 | 12 | 4 | NA | 0.1 | walk | 11 | + | + | + | + | - | NA | - | - | 120 | NA | - |
| P51 | M-P | m | 10.1 | 10.5 | 1.2 | - | 4 | walk | 4 | + | + | - | - | - | + | - | - | NA | NA | - |
| P52 | M-P | m | 5.5 | 5.9 | 1.5 |  | 1.5 | walk | 3.5 | + | + | - | - | - | - | - | - | 115 | myo | - |
| P53 | E-S | f | 0.3 | 0.3 | NP | + | 0.1 | NO | NA | + | + | - | + | + | - | - | + | nor | myo | - |
| P54 | Mild | m | 4.1 | 13 | NA | - | 4.1 | walk | 13 | + | + | - | - | - | + | - | + | 264 | neu+myo | - |
| P55 | M-P | f | 3.6 | 3.9 | 2 | - | 0.2 | walk | walking | + | + | - | + | - | + | - | - | 438 | neu+myo | - |
| P56 | M-P | m | 1.9 | 8 | 1.9 | - | 1.5 | walk | 8 | + | + | - | - | - | - | - | - | NA | nor | - |
| P57 | M-P | m | 3.1 | 7 | 2 | - | 0.1 | walk | 7 | + | + | - | - | - | - | - | - | 600 | neu | - |
| P58 | M-P | m | 6 | 6 | 1.5 | - | 1.5 | walk | walking | - | NA | + | + | - | NA | + | NA | 44 | neu | - |
| P59 | Mild | f | 7 | 16 | 1.5 | - | 3 | walk | 16 | - | + | - | + | - | + | + | + | 119 | myo | PO |
| P60 | BM | f | 12.5 | 18 | 1 | - | 1.5 | walk | walking | - | - | - | - | + | + | - | + | 357 | NA | AT |
| P61 | Mild | m | 3.3 | 9.3 | 1.3 | - | 3.3 | walk | walking | NA | + | - | - | - | NA | NA | NA | 596 | neu+myo | - |
| P62 | M-P | M | 6 | 12 | 1.5 | + | 0.1 | walk | 8 | + | + | - | - | + | + | - | - | 218 | myo | - |
| P63 | M-P | M | 5 | 10 | 2 | - | 0.2 | walk | 10 | + | + | + | + | + | + | - | - | 96 | myo | - |
| P64 | Mild | F | 17 | 17 | 2 | - | 1.5 | walk | 16 | + | + | + | - | - | + | + | - | 119 | myo | - |
| P65 | BM | F | 25 | 26 | 5 | - | NA | walk | walking | - | - | - | - | - | + | - | - | 492 | myo | - |
| P66 | Mild？ | m | 5 | 5 | NA | - | 3 | walk | walking | - | + | - | - | - | - | - | - | 557 | myo | - |
| P67 | M-P | f | 2.5 | 2.5 | 2 | - | 0.1 | walk | walking | + | + | - | + | - | - | - | - | 4000 | NA | DDH |
| P68 | BM | m | 12 | 12 | 1 | - | 1.5 | run | walking | + | - | + | - | - | + | + | + | 210 | myo+neu | - |
| P69 | M-P | f | 7.7 | 7.7 | 2 | - | 0.5 | walk | walking | - | + | + | + | - | - | - | - | 333 | myo | - |
| P70 | Mild | m | 13 | 13 | 1 | - | 4 | walk | walking | - | - | - | - | - | + | - | - | 725 | nor | - |
| P71* | M-P | m | 4 | 4 | 1.5 | - | 1 | walk | walking | - | + | - | - | - | - | - | - | 107 | myo | - |
| P72* | M-P | f | 0.2 | 3.5 | 1.5 | - | 0.1 | walk | walking | NA | + | NA | NA | + | NA | - | + | 543 | myo | Tor |
| P73* | M-P | m | 3.9 | 4 | 1.7 | - | 2 | walk | walking | NA | NA | NA | NA | NA | NA | NA | NA | NA | ND | - |
| P74* | M-P | f | 5 | 5 | 1.5 | - | 5 | walk | 5 | NA | NA | NA | NA | NA | NA | NA | NA | 318 | myo | - |
| P75* | BM | m | 11.6 | 20 | NA | - | 11.6 | walk | walking | NA | NA | NA | NA | NA | NA | NA | NA | 398 | myo | - |
| P76* | Mild？ | m | 4.8 | 9.5 | NA | - | 4.8 | walk | walking | NA | NA | NA | NA | NA | + | NA | NA | 87 | myo | - |
| P77* | E-S | m | 1 | 1.1 | NP | + | 0.1 | sit | NA | - | + | - | + | - | - | - | - | 95 | myo | - |
| P78* | M-P | m | 2.3 | 9 | 2.3 | - | 1 | walk | 9 | - | + | - | + | - | - | - | - | 345 | neu | - |
| P79* | M-P | m | 7.3 | 7.3 | 2 | - | 0.1 | walk | 6 | - | + | - | - | - | - | - | - | 471 | myo+neu | - |
| P80* | M-P | m | 3.3 | 3.3 | 2.3 | - | 0.1 | walk | walking | + | + | - | - | - | + | + | + | NA | myo+neu | - |
| P81* | M-P | m | 6.5 | 7 | 1.5 | - | 0.1 | walk | walking | - | + | - | - | - | + | NA | NA | 325 | neu | - |
| P82* | E-S | f | 5.5 | 6.5 | NP | + | 0.5 | sit | NA | - | + | 5.5 | - | + | - | - | - | 75 | nor | - |

Legend: E-S: early-severe UCMD; M-P: moderate-progressive UCMD; Mild: mild UCMD; BM: bethlem myopathy; F: female; M: male; CK: creatine kinase; EMG: electromyography; NA: not available; Nor: normal; NP: not applicable; myo: myopathic; neu: neuropathic; Tor: surgery of torticollis; AT: release of Achilles's tendon; MO: multilevel orthopedic surgery; PO: pelvic osteotomy; patients with * indicated that genetic tests were not done or available.

S-Table 2. Genotype spectrum of 70 collagen VI-related myopathy patients

| No. | phenotype | Gene | Exon | Domain | Nucleotide Change | Hom/Het | Amino Acid Change | Mutation type | Novelty | reported phenotype | Reference/PMID | origin | inheritance pattern | FH |
| --- | --- | --- | --- | --- | --- | --- | --- | --- | --- | --- | --- | --- | --- | --- |
| P1 | Mild | COL6A1 | Ex12 | THD | c.932G>A | het | p.G311D | missense | reported | Interm | 24271325 | de novo | sporadic | - |
| P2 | M-P | COL6A1 | Ex10 | THD | c.868G>A | het | p.G290R | missense | reported | UCMD | 15689448 | de novo | sporadic | - |
| P3 | Mild | COL6A1 | In13 | THD | c.1003-2A>G | het | / | splicing | reported | SSCD | 25635128 | de novo | sporadic | - |
| P4 | M-P | COL6A1 | In1 | N | c.97+1G>A | hom | / | splicing | novel | / | / | NA | AR | - |
| P5 | Mild | COL6A1 | In14 | THD | c.1056+5G>A | het | p.V2093A fs*11 | splicing | reported | BM | 29419890 | de novo | sporadic | - |
| P6 | M-P | COL6A1 | Ex9 | THD | c.850G>A | het | p.G284R | missense | reported | UCMD/BM | 15689448 | de novo | sporadic | - |
| P7 | M-P | COL6A1 | Ex10 | THD | c.868G>A | het | p.G290R | missense | reported | UCMD | 15689448 | de novo | sporadic | - |
| P8 | BM | COL6A1 | In14 | THD | c.1056+1G>A | het | / | splicing | reported | BM | 10419498 | de novo | sporadic | - |
| P9 | BM | COL6A1 | In14 | THD | c.1056+1G>C | het | / | splicing | reported | undefined | 29382405 | NA | sporadic | - |
| P10 | M-P | COL6A1 | In13 | THD | c.1003-2A>T | het | / | splicing | novel | / | / | maternal | AD | + |
| P11 | E-S | COL6A1 | Ex11 | THD | c.904G>A | het | p.G302R | missense | reported | UCMD | 24271325 | NA | sporadic | - |
| P12 | M-P | COL6A1 | Ex10 | THD | c.877G>A | het | p.G293R | missense | reported | BM | 20976770 | de novo | sporadic | - |
| P13 | M-P | COL6A1 | In14 | THD | c.1056+1G>A | het | / | splicing | reported | BM | 10419498 | maternal | AD | + |
| P14 | M-P | COL6A1 | Ex11 | THD | c.904G>A | het | p.G302R | missense | reported | UCMD | 24271325 | paternal | AD | * |
| P15 | Mild | COL6A1 | Ex11 | THD | c.913G>T | het | p.G305W | missense | novel | / | / | maternal | AD | * |
| P16 | Mild | COL6A1 | Ex10 | THD | c.859G>A | het | p.G287R | missense | reported | Interm | 24038877 | de novo | sporadic | - |
| P17 | BM | COL6A1 | Ex10 | THD | c.877G>A | het | p.G293R | missense | reported | BM | 20976770 | NA | sporadic | - |
| P18 | M-P | COL6A1 | In10 | THD | c.904-5T>G | het | / | splicing | novel | / | / | de novo | sporadic | - |
| P19 | M-P | COL6A1 | E12 | THD | c.957G>A | het | p.K319K | splicing | novel | / | / | de novo | sporadic | - |
| P20 | M-P | COL6A1 | Ex9 | THD | c.814G>C | het | p.G272R | missense | novel | / | / | de novo | sporadic | - |
| P21 | Mild | COL6A1 | In21 | THD | c.1461+4A>G | het | / | splicing | reported | UCMD | 24271325 | paternal | AD | * |
| P22 | M-P | COL6A1 | Ex9 | THD | c.815G>A | het | p.G272D | missense | reported | BM | 15955946 | de novo | sporadic | - |
| P23 | BM | COL6A1 | Ex14 | THD | c.1056+1delG | het | / | splicing | reported | undefined | 25635128 | de novo | sporadic | - |
| P24 | M-P | COL6A1 | Intron11 | THD | c.930+189C>T | het | / | splicing | reported | / | / | de novo | sporadic | - |
| P25 | M-P? | COL6A1 | Ex10 | THD | c.877G>A | het | p.G293R | missense | reported | BM | 20976770 | NA | sporadic | - |
| P26 | BM | COL6A1 | Ex8 | THD | c.788G>A | het | p.G263D | missense | Novel | BM | / | de novo | sporadic | - |
| P27 | M-P | COL6A1 | In12 | THD | c.957+1G>A | het | p.G311_K319del | splicing | reported | UCMD | 15563506 | NA | sporadic | - |
| P28 | BM | COL6A1 | Ex12 | THD | c.956A>G | het | p.K319R | missense | reported | / | / | maternal | AD | * |
| P29 | M-P | COL6A2 | Ex24 | C1 | c.1816G>C | hom | p.E591Tfs*/splicing | PTC/splicing | reported | UCMD | 29419890 | NA | AR | - |
| P30 | BM | COL6A2 | Ex26 | C1 | c.2093C>T | het | p.A698V | missense | reported | BM/SSCD | 34167565 | NA | sporadic | - |
| P31 | BM | COL6A2 | In4 | THD | c.736-1G>T | het | p.C246_K267del | splicing | novel | / | / | NA | sporadic | - |
| P32 | Mild？ | COL6A2 | Ex7 | THD | c.883G>A | Het | p.G295R | missense | novel | / | / | NA | sporadic | - |
| P33 | M-P | COL6A2 | Ex7 | THD | c.875G>A | het | p.G292D | missense | reported | Interm | 24038877 | de novo | sporadic | - |
| P34 | BM | COL6A2 | Exon25 | C1 | c.1870G>A | het | p.E624K | missense | reported | undefined | 34167565 | maternal | AD | + |
| P35 | M-P | COL6A2 | In9 | THD | c.954+1G>A | het | / | splicing | novel | / | / | de novo | sporadic | - |
| P36 | M-P | COL6A2 | In9 | THD | c.954+1G>A | het | / | splicing | novel | / | / | de novo | sporadic | - |
| P37 | M-P | COL6A2 | In9 | THD | c.955-1G>C | het | / | splicing | novel | / | / | de novo | sporadic | - |
| P38 | Mild | COL6A2 | In4 | N1 | c.736-2A>C | het | / | splicing | novel | / | / | de novo | sporadic | - |
| P39 | M-P | COL6A2 | In5 | THD | c.801+2T>G | het | / | splicing | novel | / | / | de novo | sporadic | - |
| P40 | M-P | COL6A2 | Ex28 | C2 | c.2817C>T | het | p.H939H | missense | novel | / | / | NA | sporadic | - |
| P41 | Mild | COL6A2 | E11 | THD | c.1037G>A | hom | p.G346E | missense | novel | / | / | maternal | AR | + |
| P42 | Mild | COL6A2 | E11 | THD | c.1037G>A | hom | p.G346E | missense | novel | / | / | maternal | AR | + |
| P43 | M-P | COL6A2 | In5 | THD | c.801+1G>A | het | / | splicing | reported | BM | 29406609 | maternal | AD | + |
| P44 | M-P | COL6A2 | Ex7 | THD | c.884G>A | het | p.G295E | missense | novel | / | / | NA | Sporadic? | - |
| P45 | M-P | COL6A2 | Ex6 | THD | c.812G>A | het | p.G271D | missense | reported | SSCD/UCMD | 17785673 | NA | Sporadic? | - |
| P46 | Mild？ | COL6A2 | Ex25 | C1 | c.1873A>C | het | P.S625R | missense | novel | / | / | paternal | AD | + |
| p47 | Mild？ | COL6A2 | Ex22 | C1 | c.1689delT | het | p.G564Vfs*32 | deletion | novel | / | / | paternal | AR | - |
|  |  |  | Ex28 | C2 | c.2739-2741del | het | p.914Fdel | deletion | novel | / | / | maternal | AR | - |
| P48 | M-P | COL6A2 | Ex6 | THD | c.803G>A | het | p.G268D | missense | novel | / | / | de novo | sporadic | - |
| P49 | BM | COL6A2 | In17 | THD | c.1458+1_c.1458+4del | het | p.G466_ E486del | deletion | reported | undefined | 18378883 | NA | AR? | - |
|  |  |  | Ex28 | C2 | c.3006dupT | het | p.D1003* | insertion | Novel | / | / | NA | AR? | - |
| P50 | E-S | COL6A2 | Ex6 | THD | c.812G>A | het | p.G271D | missense | reported | SSCD/UCMD | 17785673 | NA | sporadic? | - |
| P51 | M-P | COL6A2 | E12 | THD | c.1096C>T | het | p.R366X | nonsense | reported | UCMD | 19309692 | paternal | AR | - |
|  |  |  | Ex25 | C1 | c.1998_1999del | het | p.S66Rfs*5 | frame shift | novel | / | / | maternal | AR | - |
| P52 | M-P | COL6A2 | Ex28 | C2 | c.2515_2516delGA | Hom | p.D839Rfs*7 | frame shift | novel | / | / | parental | AR | - |
| p53 | E-S | COL6A2 | Ex14 | THD | c.1242_1243del | het | p.G415Afs*15 | frame shift | novel | / | / | paternal | AD | * |
| P54 | Mild | COL6A3 | Ex9 | N4 | c.4103C>T | het | p.T1368M | missense | reported | LGMD | 29970176 | paternal | AD | * |
| P55 | M-P | COL6A3 | In16 | THD | c.6210+1G>A | het | / | splicing | reported | UCMD | 15563506 | NA | sporadic | - |
| P56 | M-P | COL6A3 | In17 | THD | c.6283-2A>G | het | / | splicing | novel | / | / | de novo | sporadic | - |
| P57 | M-P | COL6A3 | Ex17 | THD | c.6276-6282del | het | p.V2093A fs*11 | deletion | novel | / | / | de novo | sporadic | - |
| P58 | M-P | COL6A3 | Ex11 | N2 | c.4912G>A | het | p.A1638T | missense | reported | Interm | 24038877 | NA | sporadic | - |
| P59 | M-P | COL6A3 | In16 | THD | c.6210+1G>A | het | / | splicing | reported | UCMD | 15563506 | NA | sporadic | - |
| P60 | BM | COL6A3 | Ex2 | SP | c.74C>A | het | p.A25D | missense | novel | / | / | NA | sporadic | - |
| P61 | Mild | COL6A3 | Ex16 | THD | c.6206G>T | het | p.G2068C | missense | novel | / | / | maternal | AD | + |
| P62 | M-P | COL6A3 | In18 | THD | c.6309+3A>G | het | p.G2095_K2103 del | splicing | reported | UCMD | 20976770 | NA | sporadic | - |
| P63 | M-P | COL6A3 | In16 | THD | c.6210+1G>T | het | p.G2053_P2070del | splicing | reported | UCMD | 20976770 | NA | sporadic | - |
| P64 | M-P | COL6A3 | Ex18 | THD | c.6302G>C | het | p.G2101A | missense | novel | / | / | maternal | AD | + |
| P65 | BM | COL6A3 | In15 | THD | c.6156+1G>A | Het | / | splicing | reported | UCMD | 15689448 | maternal | AD | + |
| P66 | Mild？ | COL6A3 | Ex17 | THD | c.6220G>C | het | p.G2074R | missense | novel | / | / | de novo | sporadic | - |
| P67 | M-P | COL6A3 | In16 | THD | c.6210+1_6210+17del | het | / | deletion | novel | / | / | de novo | sporadic | - |
| P68 | BM | COL6A3 | In19 | THD | c.6354+1G>A | het | / | splicing | reported | BM | 28688748 | paternal | AD | + |
| P69 | M-P | COL6A3 | Ex17 | THD | c.6212G>A | het | p.G2071D | missense | reported | UCMD-BM | 28688748 | de novo | sporadic | - |
| P70 | Mild | COL6A3 | Ex12 | N1 | c.5525G>A | het | p.G1842E | missense | reported | BM | 25535305 | paternal | AD | + |

Legend: CD: complete deficiency; SSCD: sarcolemma-specific collagen VI deficiency; Ex: exon; In: intron; THD: triple helical domain; C1:the first C-terminal globular domain; C2:the second C-terminal globular domain; N1:the first N-terminal globular domain; SP: signal peptide Homo: homogeneous; het: heterogeneous; PTC: premature termination codon-causing; NA: not available; ND: not determined; FH: family history; SP: signal peptide; * asymptomatic carrier or somatic mosaic.
